# Quantifying Leaf Trait Covariations and Their Relationships with Plant Adaptation Strategies along an Aridity Gradient

**DOI:** 10.3390/biology10101066

**Published:** 2021-10-19

**Authors:** Yanzheng Yang, Le Kang, Jun Zhao, Ning Qi, Ruonan Li, Zhongming Wen, Jalal Kassout, Changhui Peng, Guanghui Lin, Hua Zheng

**Affiliations:** 1 State Key Laboratory of Urban and Regional Ecology, Research Center for Eco-Environmental Sciences, Chinese Academy of Sciences, Beijing 100085, China; yangyzh@rcees.ac.cn (Y.Y.); rnli@rcees.ac.cn (R.L.); 2 Ministry of Education Key Laboratory for Earth System Modeling, Department of Earth System Science, Tsinghua University, Beijing 100084, China; lingh@tsinghua.edu.cn; 3 East China Inventory and Planning Institute of the State Administration of Forestry and Grassland, Hangzhou 310019, China; hdybhc@163.com; 4 China Aero Geophysical Survey & Remote Sensing Center for Natural Resources, Beijing 100083, China; zhaojun01@mail.cgs.gov.cn; 5 School of Information Science & Technology, Beijing Forestry University, Beijing 100083, China; ningqi830@163.com; 6 Institute of Soil and Water Conservation, Chinese Academy of Sciences and Ministry of Water Resources, Yangling 712100, China; zmwen@ms.iswc.ac.cn; 7 Laboratory of Applied Botany, BioAgrodiversity Team, Faculty of Sciences, Abdelmalek Essaadi University, Tetouan 93002, Morocco; jalalkassout@gmail.com; 8 Department of Biological Sciences, Institute of Environmental Sciences, University of Quebec at Montreal, Montréal, QC H3C 3P8, Canada; peng.changhui@uqam.ca; 9 College of Resources and Environment, University of Chinese Academy of Sciences, Beijing 100049, China

**Keywords:** plant adaptation strategy, plant functional traits, trait covariation, multivariate analysis, aridity gradient

## Abstract

**Simple Summary:**

Plants usually adopt different strategies to adapt to their surrounding environments. Accurately quantifying plant strategies is of great interest in trait-based ecology, in particular to understand the responses of ecological structures and processes. In the last two decades, these strategies have been described qualitatively; however, the use of quantitative methods is still lacking. In this study, we used a plant functional trait approach to discuss plant strategies along an aridity gradient. We found that eight functional traits divided into four dimensions represent four adaptation strategies: energy balance, resource acquisition, resource investment and water use efficiency. We also concluded that climate and soil together with family (vegetation succession) were the main driving forces of trait covariations. Our study provided a new perspective to understand plant functional responses to aridity gradients, which is helpful for ecological management and vegetation restoration programs in arid regions.

**Abstract:**

A trait-based approach is an effective way to quantify plant adaptation strategies in response to changing environments. Single trait variations have been well depicted before; however, multi-trait covariations and their roles in shaping plant adaptation strategies along aridity gradients remain unclear. The purpose of this study was to reveal multi-trait covariation characteristics, their controls and their relevance to plant adaptation strategies. Using eight relevant plant functional traits and multivariate statistical approaches, we found the following: (1) the eight studied traits show evident covariation characteristics and could be grouped into four functional dimensions linked to plant strategies, namely energy balance, resource acquisition, resource investment and water use efficiency; (2) leaf area (LA) together with traits related to the leaf economic spectrum, including leaf nitrogen content per area (*N*_area_), leaf nitrogen per mass (*N*_mass_) and leaf dry mass per area (LMA), covaried along the aridity gradient (represented by the moisture index, MI) and dominated the trait–environmental change axis; (3) together, climate, soil and family can explain 50.4% of trait covariations; thus, vegetation succession along the aridity gradient cannot be neglected in trait covariations. Our findings provide novel perspectives toward a better understanding of plant adaptations to arid conditions and serve as a reference for vegetation restoration and management programs in arid regions.

## 1. Introduction

Aridity acts as a strong environmental filter for plant survival, growth and development and therefore has considerable effects on community structure and ecosystem functions, including primary productivity and nutrient cycling [1,2]. For instance, aridity favors species with small leaves as a part of their adaptation strategy to adapt soil to water deficits and nutrient limitations [3]. Plants undergo environmental adaptation and evolve an optimal phenotype, forming a set of adaptation strategies at both the community and individual levels [4]. To date, researchers have usually ascribed the single axis of a plant’s adaptation strategy to environmental changes, but strategies explained by covariation and a tradeoff in traits related to nutrient acquisition and conservation have been neglected [5,6]. These strategies can be quantified by the covariation of several plant functional traits, which are any morpho-physio-phenological traits that have an impact on plant growth, reproduction and survival [7,8]. From the perspective of trait-based ecology, more information on plant adaptation to aridity gradients is needed.

Functional trait covariation may reflect plant strategies to stressful aridity conditions. In fact, variability in environmental conditions is considered as a filter of traits [9,10]; therefore, variations in plant functional traits reflect shifts in plant adaptation strategies under changing environmental conditions. For example, changes in plant height, leaf mass per area (LMA) and leaf nitrogen content per area (*N*_area_) are directly linked to resource acquisition and drought tolerance, which have been widely reported [8,11,12]. Meanwhile, plants in resource-poor environments prone to arid conditions are expected to favor conservative trait syndromes, such as slower growth rates, lower production and higher root density [13]. Within arid sites, C_3_ plants show higher values of leaf ^13^C, which reflect higher water use efficiency [14]. Thus, it has been reported that plants balance their investment in either enzymatic reactions or electronic transport in the process of photosynthesis to avoid wasting resources [15].

Several studies have successfully quantified plant adaptation to arid environments [1,14,16]; nonetheless, only a few of them have yielded results that consider the interpretation difficulty of trait covariation and their controls. Some issues remain to be resolved:

(1) Traits do not vary independently but show covariation and tradeoff relationships [17]. However, from the perspective of plant functional traits, plant strategies adopted to simultaneously balance conservation and resource acquisition remain unclear.
(2) Climate and soil are widely recognized to control trait variation [18,19,20]. However, the effects of climate and soil intersections have not been fully quantified, especially under arid conditions.
(3) Vegetation succession along an aridity gradient resulted in the replacement of larger leaf plants by plants with small and high-efficiency leaves [21]. The contribution of vegetation distribution (family) to trait covariations is still unknown along aridity gradients.

In the past few decades, the Loess Plateau in China has implemented many large-scale ecological protection and restoration projects [22,23]. The observed regional climate changes (especially in precipitation trends) has caused great effects on ecosystem composition and functions [24]. Therefore, quantifying plant adaption to regional climate changes, especially in arid areas, is a matter of urgency. In this study, taking the Loess Plateau as the study area and using multivariate statistics, we aim to (1) quantify the covariation characteristics of eight functional traits and their representative dimensions (adaptation strategies); (2) reveal trait variation along the precipitation and temperature gradients; and (3) explore the controls of eight key traits along the aridity gradient on the Loess Plateau. Hence, the aim of this study is to reveal the adaptation strategies of plants to aridity gradients throughout quantifying the covariation of eight fictional traits.

## 2. Materials and Methods

### 2.1. Study Area and Sampling Strategy

**Figure 1:**
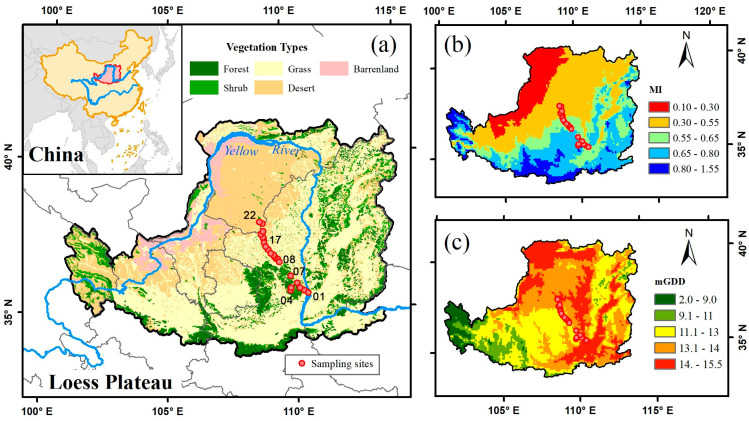
Distributions of the sampling sites across the aridity gradient on the Loess Plateau. (**a**) Vegetation distribution—the numbers represent the site IDs in Table 1. (**b**) Distribution map for moisture index (MI). (**c**) Distribution map for growing-season monthly mean temperature above 0 °C (mGDD_0_).

The Loess Plateau is a highland area located in the middle of the Yellow River basin in China (Figure 1a) and is prone to serious soil and water loss [25]. With an area of more than 620,000 km^2^, this region features a temperate continental climate with a mean annual temperature ranging from 4 °C to 14 °C and annual precipitation ranging from approximately 200 mm to 800 mm, forming three distinct zones along an aridity gradient: semihumid (moisture index, MI > 0.67), semiarid (0.55 < MI ≤ 0.67) and arid (MI ≤ 0.55). From southeast to northwest, precipitation decreases on the Loess Plateau (Figure 1b). Thus, from south to north, the temperature (represented by a growing-season monthly mean temperature above 0 °C (mGDD_0_)) increases and is closely related to changes in elevation. In the past few decades, many ecological projects have been implemented on the Loess Plateau (Figure 1c), and, therefore, the effects of the local climate and plant adaptation to environmental changes have been widely studied.

Eight morphological, chemical and photosynthetic traits were measured and sampled from 105 species and 271 records along 22 sites in August 2016 and 2017 (Figure 1a, Table 1 and Table A1). The 105 species were grouped into 38 families, and the main families are described in Table A2. The sampling procedure allows one to cover all of the vegetation types and climate gradients of the Loess Plateau, which represents an ideal design to reveal plant responses and functional strategies to aridity gradients. Sampling was restricted to dominant species and communities with no clear human disturbance. Thus, along the sampling transect, the vegetation types changed from forestland to shrubland and desert, with noticeable vegetation succession, making it ideal to study changes in ecological structure and functions from the perspective of plant functional traits.

### 2.2. Data Descriptions of Traits, Climate and Soil

All plots were located on sunny slopes, and middle-aged plants were sampled. Eight leaf functional traits, namely leaf area (LA), leaf nitrogen content (*N*_mass_), *N*_area_, LMA, leaf dry mass content (LDMC), maximum carboxylation rate standardized to 25 °C (*V*_cmax25_), maximum electron transport rate standardized to 25 °C (*J*_max25_) and the ratio of intercellular to ambient CO_2_ concentration (*c*_i_:*c*_a_, termed *χ* in this study), which could capture most plant functions, were measured. All traits were measured following standardized protocols, as proposed by Cornelissen et al. [26]. LA is the projected area of a leaf (or leaflet for compound leaves), which is linked to the leaf energy balance [27]. LDMC is the oven-dry mass divided by its fresh mass. LMA is the oven-dry mass divided by fresh LA, which is the inverse of specific leaf area (SLA) and highly related to the resistance of plants to both herbivory and drought [28]. *N*_mass_ is the leaf nitrogen per mass, and *N*_area_ is the leaf nitrogen per area, which are two indicators of photosynthetic capacity [29]. LDMC, LMA, *N*_mass_ and *N*_area_ are key traits in the leaf economics spectrum (LES), which reflects a plant’s investment of nutrients along the acquisition–conservation continuum [30,31].

*V*_cmax_ and *J*_max_ were calculated using a one-point method from the light-saturated rate of net CO_2_ fixation at ambient CO_2_ (*A*_sat_) and the light-saturated rate of net CO_2_ fixation at high CO_2_ (*A*_max_) [32]. *V*_cmax_ and *J*_max_ were standardized to 25 °C following the proposed methods of Niinemets et al. [33]. From the 272 records, 46 records for *V*_cmax25_ and *J*_max25_ were not measured, and, therefore, we used a multilayer perceptron (MLP) [34], which is a class of feedforward artificial neural networks with high simulation accuracy. In this experiment, the training and validation accuracy for *V*_cmax25_ and *J*_max25_ reached 0.55. *χ* was converted from leaf δ^13^C measurements after eliminating the effects of sampling year and latitude, following the method proposed by Cornwell et al. [35]. *χ* is a useful indicator in aridity areas because it determines the balance between carbon gain and water loss, which is closely regulated by the stomata and reflects the adaptation of plants in arid conditions.

The climate of each site was defined using three climate variables, namely growing degree days above 0 °C (mGDD_0_), monthly accumulated photosynthetically active radiation during the thermal growing season (mPAR_0_) and the ratio of mean annual precipitation to annual equilibrium evapotranspiration (moisture index, MI). MI is a widely recommended indicator of aridity stress, which is the inverse of dry index and can reflect the plant-available water content in the soil. These bioclimatic variables were calculated from observations retrieved from meteorological stations on the Loess Plateau. Two variables representing soil conditions for optimal plant growth were selected, namely soil organic matter (SOM) and soil pH at 0~30 cm depth.

### 2.3. Multivariate Statistical Analysis

Principal component analysis (PCA) and redundancy analysis (RDA) were used to analyze trait covariation and to reveal the relationships between traits, considering traits as response variables and climate/soil/family variables as predictors [36,37]. PCA and RDA were performed with the *vegan* package in R language [38]. LA was square-root transformed to create a linear relationship. Calculated using the method of variation partitioning, a Venn diagram was used to show the contributions of the climate, soil and family, and their independent and intersection effects were explicitly considered [39]. The taxonomic rank at the family level was used in forward analysis looking at the shared evolutionary history between species and the assumed similarities in their responses to environmental changes. Thus, for the purpose of our study, it was more suitable to use the family level rather than the genera or species in the statistical analysis. Ordinary linear regression was employed to describe the variation in a single trait response to the climate gradient, and the 95% confidence intervals are shown.

## 3. Results

### 3.1. Trait Covariation and Corresponding Adaptation Strategies

**Figure 2:**
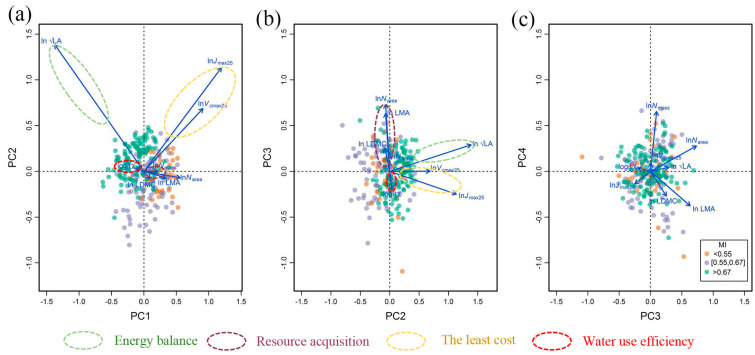
Principal component (PC) patterns of eight plant functional traits. (**a**) PC1 versus PC2. (**b**) PC2 versus PC3. (**c**) PC3 versus PC4. LA: leaf area; *N*_mass_: leaf nitrogen content; *N*_area_: leaf nitrogen per area; LMA: leaf mass per area; LDMC: leaf dry mass content; *V*_cmax25_: maximum carboxylation rate standardized to 25 °C; *J*_max25_: maximum electron transport rate standardized to 25 °C; *χ*: ratio of intercellular to ambient CO_2_ concentration.

The PCA analysis that included all plants and sites confirmed our hypothesis that the eight traits studied are not independent but show covariation patterns (Figure 2, Table 2). The first three axes captured 84.32% of the total variation in the eight traits. The first axis captured 41.13% of variation, which was mainly controlled by LA, *N*_area_, *V*_cmax25_ and *J*_max25_. The second axis accounted for 32.61% of variation and was dominated by LA, *V*_cmax25_ and *J*_max25_. The third axis accounted for 10.58% of variation and was dominated by *N*_area_ and LMA. Both LA and *χ* changed independently and did not covary with the other traits (Figure 2). *V*_cmax25_ and *J*_max25_ closely covaried in the first two PCA axes. *N*_mass_, *N*_area_, LMA and LMDC, four important traits of LES, also presented clear covariation characteristics, especially on the second axis (capturing 32.61% of the total variations), which show similar weights to the first axis.

**Figure 3:**
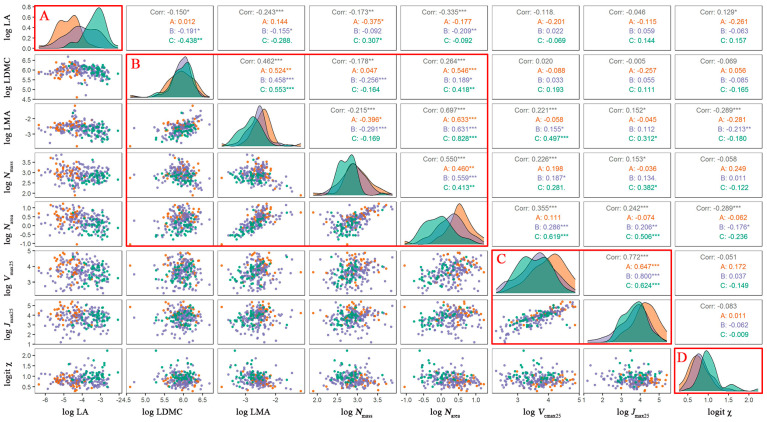
Correlations among eight plant functional traits. (**A**–**D**) Four trait dimensions of eight traits. LA: leaf area; *N*_mass_: leaf nitrogen content per mass; *N*_area_: leaf nitrogen per area; LMA: leaf mass per area; LDMC: leaf dry mass content; *V*_cmax25_: maximum carboxylation rate standardized to 25 °C; *J*_max25_: maximum electron transport rate standardized to 25 °C; *χ*: ratio of intercellular to ambient CO_2_ concentration. “*” indicates *p* < 0.05. “**” indicates *p* < 0.01. “***” indicates *p* < 0.001.

The correlation matrix (Figure 3) confirmed our results obtained from the PCA analysis that the eight studied traits are organized into four trait dimensions. The first dimension was LA, which had a low correlation with other traits and represented plant behavior during evaporation (energy balance). The second dimension was composed of LDMC, LMA, *N*_area_, and *N*_mass_, which were closely related within this dimension and weakly related to traits in other dimensions. This dimension is linked to a plant’s strategy for resource acquisition. *V*_cmax25_ and *J*_max25_ constituted the third dimension, with a correlation coefficient reached at 0.77. In this dimension, *V*_cmax25_ and *J*_max25_ varied tightly because plants should not invest more in either photosynthesis or electronic transport to maintain a lower energy cost. The fourth dimension was *χ*, which was found to be unrelated to the other traits. This dimension is directly linked with water use efficiency. The clear color differences of points in the histogram (Figure 3) and scatter plots (Figure 2a,b) indicate that trait covariations were not random but presented significant variation along the precipitation gradient.

### 3.2. Trait Variations along the Aridity Gradient (MI Decreased)

**Figure 4:**
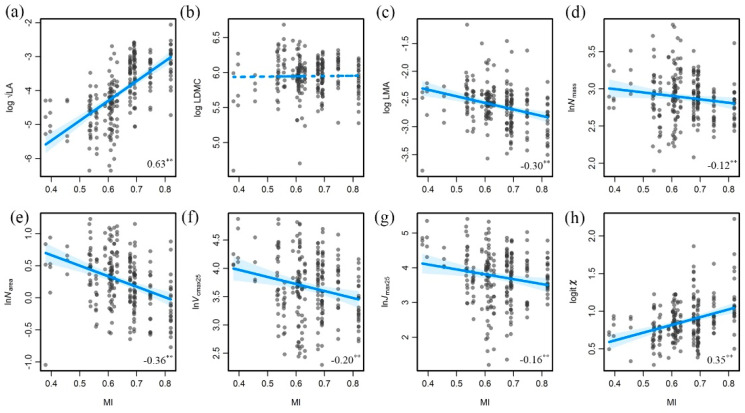
Trait variations along the aridity gradient (decreasing moisture index, MI). (**a**) LA versus MI. (**b**) LDMC versus MI. (**c**) LMA versus MI. (**d**) *N*_mass_ versus MI. (**e**) *N*_area_ versus MI. (**f**) *V*_cmax25_ versus MI. (**g**) *J*_max25_ versus MI. (**h**) *χ* versus MI. The bottom right numbers are the correlation coefficients and their significance, with “**” indicating *p* < 0.01. LA: leaf area; *N*_mass_: leaf nitrogen content per mass; *N*_area_: leaf nitrogen per area; LMA: leaf mass per area; LDMC: leaf dry mass content; *V*_cmax25_: maximum carboxylation rate standardized to 25 °C; *J*_max25_: maximum electron transport rate standardized to 25 °C; *χ*: ratio of intercellular to ambient CO_2_ concentration.

Except LDMC, all the studied traits showed significant variation trends along the aridity gradient (decreasing MI) (Figure 4). LA increased along with an increase in MI, which is consistent with the transition of vegetation types from forests (large leaf areas) to grasslands and deserts (small leaf areas). LDMC was not sensitive to moisture changes. Plants tended to have higher LMA values when facing arid conditions because of its construction cost. Both *N*_mass_ and *N*_area_ increased significantly along the aridity gradient and were related to leaf construction and photosynthetic capacity. *V*_cmax25_ and *J*_max25_ were directly linked with photosynthetic capacity, exhibiting an increase along the aridity gradient (decreasing MI), because the growth period was shorter in the arid area than in the humid area, and they required more photosynthesis efficiency to recover construction cost. *χ* decreased along the aridity gradient, which reflected an adjustment in plant stomata in arid conditions to maintain high water use efficiency.

**Figure 5:**
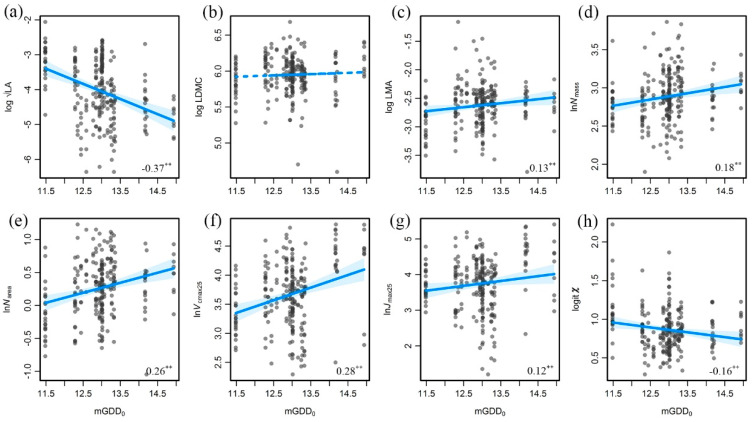
Trait variations along the temperature gradient (growing-season monthly mean temperature above 0°C, mGDD_0_). (**a**) LA versus mGDD_0_. (**b**) LDMC versus mGDD_0_. (**c**) LMA versus mGDD_0_. (**d**) *N*_mass_ versus mGDD_0_. (**e**) *N*_area_ versus mGDD_0_. (**f**) *V*_cmax25_ versus mGDD_0_. (**g**) *J*_max25_ versus mGDD_0_. (**h**) *χ* versus mGDD_0_. The bottom right numbers are the correlation coefficients and their significance, with “**” indicating *p* < 0.01. LA: leaf area; *N*_mass_: leaf nitrogen content per mass; *N*_area_: leaf nitrogen per area; LMA: leaf mass per area; LDMC: leaf dry mass content; *V*_cmax25_: maximum carboxylation rate standardized to 25 °C; *J*_max25_: maximum electron transport rate standardized to 25 °C; *χ*: ratio of intercellular to ambient CO_2_ concentration.

Trait variation patterns were not the same along the temperature gradient (increasing mGDD_0_) and the aridity gradients (Figure 5), which were mainly affected by growth temperature and latitude. LA had a pronounced decreasing trend because of the combined effects of vegetation types and latitude, indicating that large areas have a strong ability to regulate temperature. Likewise, LDMC had no significant trend toward temperature changes. *N*_mass_ and *N*_area_ increased with an increase in temperature; thus, *N*_area_ had a higher correlation coefficient than that of *N*_mass_. *V*_cmax25_ increased with an increase in temperature, which is explained by the activity of Rubisco regulating by temperature. *J*_max25_ reflects photosynthetic electron transport ability, which was higher under warm conditions. *χ* decreased with an increase in temperature as a result of the tendency of plants to close their stomata to avoid water loss in arid areas.

### 3.3. Trait Covariations and Adaptation Strategies Related to Climate and Soil Variables

**Figure 6:**
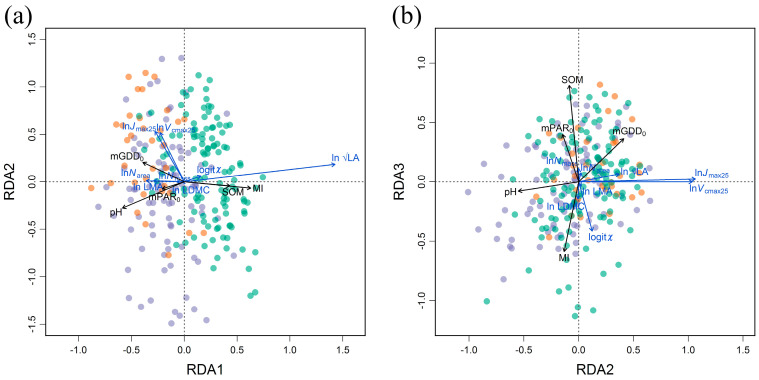
Redundancy analysis (RDA) for eight traits and climate/soil variables. (**a**) RDA1 versus RDA2. (**b**) RDA2 versus RDA3. LA: leaf area; *N*_mass_: leaf nitrogen content per mass; *N*_area_: leaf nitrogen per area; LMA: leaf mass per area; LDMC: leaf dry mass content; *V*_cmax25_: maximum carboxylation rate standardized to 25 °C; *J*_max25_: maximum electron transport rate standardized to 25 °C; *χ*: ratio of intercellular to ambient CO_2_ concentration. MI: moisture index; mGDD0; monthly growth temperature above 0 °C; mPAR0: monthly photosynthetically active radiation above 0 °C; pH: soil pH; SOM: soil organic matter content.

The highlighted four dimensions composed from the eight studied traits remained independent when filtered by climate and soil variables (Figure 6). In fact, covariations in the eight traits could be explained by the effects of climate and soil variables reaching 27.6% (Table A1), presenting distinct environmental adaptation of plants. The most important information was the covariations in LA along the MI and soil pH gradients, which dominated the first RDA axis (Figure 6a). Three traits in LES (*N*_area_, *N*_mass_ and LMA) also changed with MI changes, but LDMC was not sensitive to the first two RDA axes. The second RDA axis (RDA2) was mainly dominated by the covariations in *V*_cmax25_ and *J*_max25_ along the temperature (mGDD_0_) and soil pH gradient, which accounted for 5.2%. The third axis accounted for only 0.5% of trait covariations, which was related to *χ* variation along with MI, mGDD_0_ and mPAR_0_ (Figure 6b). Along with the climate and soil variables, the traits also showed covariation trends within four trait dimensions. For example, three traits in LES covaried along RDA1, and two photosynthetic traits (*V*_cmax25_ and *J*_max25_) covaried along RDA2.

### 3.4. Controls of Trait Covariations along the Aridity Gradient

**Figure 7:**
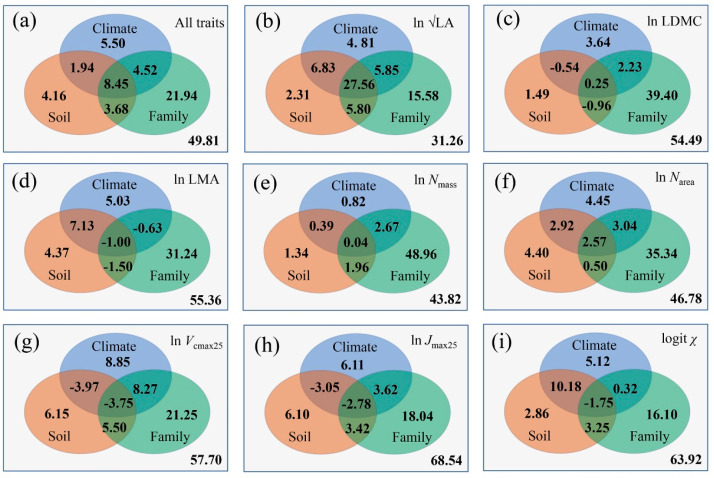
Contributions (%) of climate, soil and family to trait variations. The negative values can be treated as zero because all the values are adjusted correlation coefficients. LA: leaf area; *N*_mass_: leaf nitrogen content; *N*_area_: leaf nitrogen per area; LMA: leaf mass per area; LDMC: leaf dry mass content; *V*_cmax25_: maximum carboxylation rate standardized to 25 °C; *J*_max25_: maximum electron transport rate standardized to 25 °C; *χ*: ratio of intercellular to ambient CO_2_ concentration.

In total, 50.19% of the covariations in traits can be explained by the combination of climate, soil and family (phylogeny/vegetation succession) (Figure 7). The largest contribution comes from family, which accounted for 38.59% (Table 3). The contribution of climate to the eight trait covariations reached 20.41%, and the contribution of soil accounted for 18.23%. For LA, both climate and soil contributed considerably to its variation; however, the independent control of climate was small, and notable information was found at the intersections of climate, soil and family. For LDMC, the contribution of climate and soil was small, and family was the main determining factor of its variation. LMA was also controlled by family, and the contribution of climate and soil was small. Compared with *N*_mass_, *N*_area_ was largely controlled by climate and was less affected by family. *V*_cmax25_ and *J*_max25_ were mainly controlled by family, less than 40% originated from the three factors combined. For *χ*, the contributions of climate, soil and family ranged from 13.8% to 18.0%.

The eight studied traits had different responses to environmental changes. The intersection of climate and soil contributed 10.39% to their covariations, while family had a strong intersection with climate/soil variables (16.65%) (Figure 7a), indicating that species evolved to adapt to their changing environments along the aridity gradient. Only LA, LMA, *N*_area_ and *χ* were sensitive to aridity changes, while the contributions of other traits were less than 10%, and the most notable feature was the joint effects of family that intersected with climate/soil variables. After the effects of climate and soil were removed, variations in LDMC, LMA, *N*_mass_, *N*_area_ and *V*_cmax25_ were largely controlled by family. For LDMC, LMA, *V*_cmax25_, *J*_max25_ and *χ*, more than 50% of the variation was unexplained by the combination of climate, soil and family, which may suggest an effect of local microclimate conditions, trait–trait relationships and intrinsic factors.

## 4. Discussion

### 4.1. Importance and Significance of Studying Trait Covariations in Arid Areas

The present study illustrates the power of using large trait datasets from multiple co-existing species, spanning a broad range of climates, to reveal general patterns of trait covariations. It also shows the utility of multivariate analysis in summarizing these patterns of covariations and the research interest in variance partitioning as a means of attributing trait variability to different (and sometimes intersecting) causes of variation. However, there is a need for further extensive trait data collection, including root-related traits and isotopic measurements, thus covering an extended range of worldwide climate types.

By analyzing the covariations of eight traits along the aridity gradient, we confirmed the existence of four main dimensions of variation, which is in accordance with previous findings [40,41]. Within these dimensions, trait–trait relationships were close, but few correlations were found between them, which could mean fewer traits representing adaptation strategies, especially in the field of ecological modeling. RDA analysis presented similar dimensionality, indicating that the trait dimensions remained consistent and stable along the arid gradient. These findings have implications toward the understanding of diverse plant strategies and species diversity. LA is the first dimension and represents a plant’s strategy to maintain temperature. The second dimension is composed of LES-related traits in LES, which is an efficient indicator of quick growth and resource use. The third dimension is linked with photosynthesis, which balances construction investment and cost. The fourth dimension is *χ*, which is an indicator of water use efficiency.

We observed that the family contribution to trait covariation, which was characterized by phylogenetic replacement along the aridity gradient, was the most important determiner of trait variation. The family contribution to the eight trait covariations reached more than 38%. For LDMC, climate and soil explain only a small fraction of the trait variation; however, large variation was caused by the family factor. Therefore, predicting plant distribution changes and vegetation sensitivity under climate conditions is crucial to community and functional ecology. If the family dynamics and trait variations in each family were quantified, the family’s role and the associated mechanisms could be accurately captured [42]. This result implies that vegetation succession cannot be neglected in trait-based ecological modeling.

Apart from the family contribution to trait covariations, the considerable role of phenotypic plasticity, originating from both a genetic and environmental basis, cannot be neglected. Phenotypic plasticity is the ability of an organism or a single genotype to exhibit different phenotypes, including behavior, morphology and physiology, in different environments [43]. For instance, in this present study, the most widely distributed plant, *Lespedeza davurica*, had clear differences in leaf area along the aridity gradient, which was explained by its capacity to maintain more water in its leaves and to adapt to low temperatures. Phenotypic variability is defined as the tendency or the potential of an organism to exhibit different phenotypes under a wide range of environmental conditions, which offer an important adaptation capacity in response to environmental changes [44]. Additionally, during field sampling, efforts need to be oriented to the effects of plant age and microclimate, which could also have a significant effect on the expression of trait covariations.

### 4.2. Mechanism of Trait Covariations along the Aridity Gradient

Trait covariations occurred along the aridity gradient, and the underlying mechanisms were behind the explanation of the different plant adaptation strategies. LA is linked with the theory of energy balance [27], which means that warm and humid conditions favor plants with large leaves; in contrast, plants with small leaves are limited by the lower temperatures. Thus, variations in *N*_mass_, *N*_area_, LMA and LDMC confirm the universal nature of LES, which reflects the optimal method of balancing the construction cost and payback time of leaves [45,46]. As expected from the theory of coordination hypothesis, close covariations were seen in *V*_cmax25_ and *J*_max25_, which shows that photosynthesis depends on both carbonylation and electron transport, as leaves seek a balance when investing in these two processes to avoid energy waste [15,47]. *χ* is an indicator of CO_2_ drawdown from air to leaf, and it is described in Farquhar’s model [48] and applied in most ecological models. Hence, variation in *χ* is linked with the least-cost hypothesis, which states that leaves minimize the sum of the cost of transpiration and carboxylation [49].

### 4.3. Challenges and Future Directions

In this study, we quantified covariations in eight important traits that captured the most information on ecosystem functions and plant adaptations, and, thus, we explored their controls. However, a more in-depth analysis is needed regarding three main aspects. First, we sampled leaf functional traits, but some traits, such as root traits (specific root length, fine root density etc.) [50] and Huber values (the ratio of the sapwood cross-section and the supporting leaf area) [51] were equally important to studying plant adaptations. Second, family was listed as the most important control, but the driving mechanism and its intersection effects with climate and soil are not fully understood. Consequently, understanding the relationships between trait covariations and family would be helpful for regional ecological restoration and management programs. Third, vegetation-based models explore the incorporation of trait variations [52,53]; however, the extent and magnitude of trait variation and, thus, their covariation characteristics have not been fully quantified. In this sense, we suggest strengthening the observation of trait covariations in the field and building consistent theoretical equations for vegetation models.

We strongly suggest considering ‘functional diversity’ in ecological modeling. In fact, trait-based models can represent the co-existence of multiple trait combinations, considering the fact that this diversity provides increased community resilience when facing environmental changes [54]. The challenge is to find a generally applicable method to specify the range of proper trait combinations that is consistent with the observed patterns of trait variation within sites. Ultimately, vegetation models should be able to predict, for example, and experimentally determine the relationships between species diversity and ecosystem function [55], but this potential has yet to be realized.

## 5. Conclusions

By analyzing the covariations of eight traits, we found that plants within arid regions adopted at least four strategies related to energy balance, resource acquisition, resource investment and water use efficiency, which reflect plant adaptations to environmental changes. We also confirmed that climate, soil and family contributed to trait covariations, but the control of family in trait covariations could not be neglected. Our findings linked trait covariations with multiple adaptation strategies and provide a reference for regional ecological management and species selection in arid areas.

## Figures and Tables

**Table 1:** Characteristics of the studied sites from the China Loess Plateau transect.

Scheme	Site Name	Latitude (Degree)	Longitude (Degree)	Elevation (m)	Vegetation Types	No. of Species Samples	Moisture Index (MI)
01	Jiumen	35.64	110.58	440.3	grassland	10	0.54
02	Wangfeng	35.72	110.44	721	grassland	12	0.61
03	Qiaozigou	35.81	110.27	1219.9	forestland	24	0.69
04	Caijiachuan1	35.75	109.89	1603.2	forestland	26	0.82
05	Caijiachuan2	35.82	109.91	1244.92	forestland	20	0.75
06	Laohugou	35.97	110.16	1061.32	forestland	21	0.69
07	Zhonglousi	36.21	109.92	1087.42	forestland	32	0.68
08	Lushanmiao	36.67	109.48	1220	grassland	14	0.63
09	Houjiacun	36.77	109.42	1200	grassland	11	0.62
10	Jiugou1	36.8	109.36	1071	grassland	10	0.61
11	Jiugou2	36.8	109.36	1067	grassland	8	0.61
12	Caozhuang	36.86	109.3	1154	grassland	10	0.61
13	Liuping	36.91	109.27	1204	grassland	7	0.61
14	Caohe	36.97	109.16	1311	grassland	15	0.6
15	Fengcigeda	37.09	109.05	1434	grassland	7	0.59
16	Liandaowan	37.19	108.97	1476	grassland	10	0.56
17	Tianciwan	37.32	108.91	1592	grassland	8	0.56
18	Lugouqu	37.44	108.91	1560	grassland	8	0.53
19	Xiasandun	37.5	108.87	1584	grassland	7	0.54
20	Shuanghaize	37.69	108.87	1347	desert	4	0.46
21	Guojiazhuang	37.94	108.88	1153	desert	4	0.4
22	Batuwan	37.99	108.74	1155	desert	3	0.38

**Table 2:** Trait loading, eigenvalues and percentage of trait variation explained by the first three principal components (PCs). LA: leaf area; *N*_mass_: leaf nitrogen content; *N*_area_: leaf nitrogen per area; LMA: leaf mass per area; LDMC: leaf dry mass content; *V*_cmax25_: maximum carboxylation rate standardized to 25 °C; *J*_max25_: maximum electron transport rate standardized to 25 °C; *χ*: ratio of intercellular to ambient CO_2_ concentration.

Traits	PC1	PC2	PC3
log√LA	**−1.97 ^a^**	**1.98**	0.42
log *N*_mass_	0.31	0.00	0.14
log *N*_area_	**0.80**	−0.10	**1.05**
log LMA	0.49	−0.10	**0.90**
log LDMC	0.14	−0.12	0.36
log *V*_cmax25_	**1.30**	**0.98**	−0.00
log *J*_max25_	**1.71**	**1.62**	−0.35
logit *χ*	−0.17	0.05	−0.29
Eigenvalue	0.90	0.71	0.23
Proportion explained (%)	41.13	32.61	10.58
Cumulative proportion (%)	41.13	73.73	84.32

^a^ Values greater than 0.5 are in bold.

**Table 3:** Total contributions (%) of climate, soil and family to trait variations.

Traits	Climate (%)	Soil (%)	Family (%)
All traits	20.41	18.23	38.57
log√LA	45.05	42.50	54.79
log LDMC	5.58	0.24	40.92
log LMA	10.53	8.99	28.10
log *N*_mass_	3.92	3.73	53.63
log *N*_area_	12.98	10.38	41.44
log *V*_cmax25_	9.40	3.93	31.27
log *J*_max25_	3.90	3.69	22.30
logit *χ*	13.87	14.55	17.92

**Figure A1:**
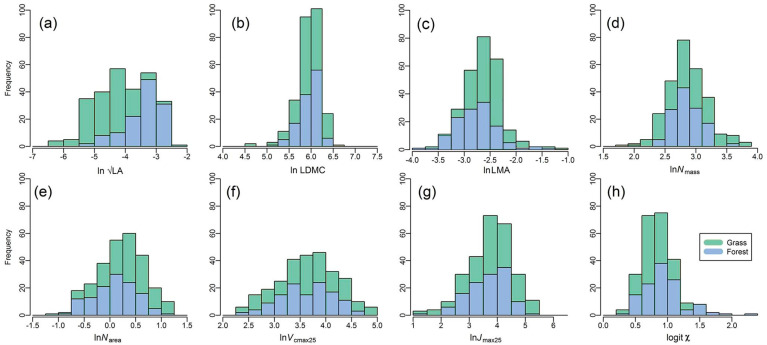
Distribution histogram of the eight studied plant functional traits, including (**a**) LA: leaf area, (**b**) LDMC: leaf dry mass content, (**c**) LMA: leaf mass per area, (**d**) *N*_mass_: leaf nitrogen content, (**e**) *N*_area_: leaf nitrogen per area, (**f**) *V*_cmax25_: maximum carboxylation rate standardized to 25 °C, (**g**) *J*_max25_: maximum electron transport rate standardized to 25 °C and (**h**) χ: ratio of intercellular to ambient CO_2_ concentration.

## Data Availability

Data are available on request due to restrictions, e.g., privacy or ethical. The data presented in this study are available on request from the corresponding author. The data are not publicly available due to the strict management of various data and technical resources within the research teams.

## Appendix A

**Table A1:** Trait loading, eigenvalues and percentage of trait variation explained by redundancy analysis (RDA) axes constrained by climate and soil variables. Values greater than 0.4 are labeled in bold.

Trait	RDA1	RDA2	RDA3	RDA4	RDA5	PC1
log√LA	2.04	0.26	0.05	0.00	0.00	−0.07
log *N*_mass_	−0.10	0.00	0.08	−0.17	0.05	−0.25
log *N*_area_	−0.51	0.02	0.06	−0.08	0.04	−0.50
log LMA	−0.41	0.02	−0.03	0.08	−0.01	−0.25
log LDMC	−0.04	−0.01	−0.10	−0.06	−0.18	−0.03
Log *V*_cmax25_	−0.33	0.74	0.00	−0.17	−0.03	−1.42
Log *J*_max25_	−0.40	0.76	0.02	0.17	0.02	−2.19
logit *χ*	0.23	0.09	−0.30	−0.04	0.08	0.09
Eigenvalue	0.47	0.11	0.01	0.01	0.00	0.68
Proportion explained (%)	21.30	5.22	0.48	0.44	0.19	31.09
Cumulative proportion (%)	21.30	26.52	27.01	27.45	27.64	58.74

**Table A2:** Description of families with a record number greater than 8.

Family	Species
Aceraceae	*Artemisia gmelinii*, *Artemisia capillaries*, *Artemisia mongolica*, *Lespedeza davurica*, *Artemisia giraldii*, *Heteropappus altaicus*, *Cirsium setosum*, *Echinops sphaerocephalus*, *Artemisia desertorum*, *Artemisia frigida*, *Artemisia sieversiana*, *Scorzonera austriaca*, *Artemisia argyi*, *Lespedeza cuneata*, *Carpesium cernuum*, *Aster tataricus*
Gramineae	*Phragmites australis*, *Bothriochloa ischaemum*, *Stipa grandis*, *Stipa bungeana*, *Cleistogenes caespitosa*, *Setaria viridis*, *Cleistogenes hancei*, *Cleistogenes chinensis*, *Leymus secalinus*, *Bromus inermis*, *Elymus dahuricus*, *Triarrhena sacchariflora*
Rosaceae	*Potentilla tanacetifolia*, *Fragaria vesca*, *Crataegus cuneata*, *Armeniaca vulgaris*, *Pyrus betulaefolia*, *Prunus salicina*, *Rosa xanthina*, *Spiraea Salicifolia*, *Cotoneaster multiflorus*, *Amygdalus davidiana*, *Rubus parvifolius*, *Pyrus betulaefolia*, *Amygdalus triloba*
Leguminosae	*Sophora davidii*, *Astragalus melilotoides*, *Thermopsis lanceolata*, *Glycyrrhiza uralensis*, *Sophora davidii*, *Oxytropis racemosa*, *Astragalus adsurgens*, *Caragana Korshinskii*, *Astragalus membranaceus*, *Lespedeza bicolor*, *Indigofera bungeana*,
Caprifoliaceae	*Lonicera japonica*, *Viburnum schensianum*, *Lonicera maackii*
Elaeagnaceae	*Hippophae rhamnoides*, *Elaeagnus umbellata*, *Elaeagnus pungens*

## References

[1] Nunes A., Köbel M., Pinho P., Matos P., de Bello F., Correia O., Branquinho C. (2017). Which plant traits respond to aridity? A critical step to assess functional diversity in Mediterranean drylands. Agric. For. Meteorol..

[2] Maracahipes L., Carlucci M.B., Lenza E., Marimon B.S., Marimon B.H., Guimaraes F.A., Cianciaruso M.V. (2018). Systematics, How to live in contrasting habitats? Acquisitive and conservative strategies emerge at inter-and intraspecific levels in savanna and forest woody plants. Perspect. Plant Ecol. Evol..

[3] Enquist B.J. (2002). Universal scaling in tree and vascular plant allometry: Toward a general quantitative theory linking plant form and function from cells to ecosystems. Tree Physiol..

[4] Ghalambor C., McKay J., Carroll S., Reznick D. (2007). Adaptive versus non-adaptive phenotypic plasticity and the potential for contemporary adaptation in new environments. Funct. Ecol..

[5] Bjorkman A., Myers-Smith I., Elmendorf S., Normand S., Rüger N., Beck P.S.A., Blach-Overgaard A., Blok D., Cornelissen J.H.C., Forbes B.C. (2018). Plant functional trait change across a warming tundra biome. Nature.

[6] Boeddinghaus R., Marhan S., Berner D., Boch S., Fischer M., Hoelzel N., Kattge J., Klaus V., Kleinebecker T., Oelmann Y. (2019). Dataset used in Boeddinghaus et al. Plant functional trait shifts explain concurrent changes in the structure and function of grassland soil microbial communities. J. Ecol..

[7] Violle C., Navas M.L., Vile D., Kazakou E., Fortunel C., Hummel I., Garnier E. (2007). Let the concept of trait be functional!. Oikos.

[8] Wright I.J., Reich P., Westoby M., Ackerly D., Baruch Z., Bongers F., Cavender-Bares J., Chapin T., Cornelissen J.H.C., Diemer M. (2004). The worldwide leaf economics spectrum. Nature.

[9] Welsh M.E., Cronin J.P., Mitchell C.E. (2016). The role of habitat filtering in the leaf economics spectrum and plant susceptibility to pathogen infection. J. Ecol..

[10] Munguía-Rosas M.A., Angulo D.F., Arceo-Gómez G., Parra-Tabla V. (2018). Variation in leaf traits across a precipitation gradient in coastal sand dunes in Yucatan Peninsula. J. Arid. Environ..

[11] Forrestel E.J., Donoghue M.J., Edwards E.J., Jetz W., du Toit J.C.O., Smith M.D. (2017). Different clades and traits yield similar grassland functional responses. Proc. Natl. Acad. Sci. USA.

[12] Yue X., Zuo X., Yu Q., Xu C., Lv P., Zhang J., Knapp A.K., Smith M.D. (2019). Response of plant functional traits of Leymus chinensis to extreme drought in Inner Mongolia grasslands. Plant Ecol..

[13] Balachowski J.A., Volaire F.A. (2018). Implications of plant functional traits and drought survival strategies for ecological restoration. J. Appl. Ecol..

[14] Prentice I.C., Meng T., Wang H., Harrison S., Ni J., Wang G. (2011). Evidence of a universal scaling relationship for leaf CO 2 drawdown along an aridity gradient. New Phytol..

[15] Maire V., Martre P., Kattge J., Gastal F., Esser G., Fontaine S., Soussana J.-F. (2012). The Coordination of Leaf Photosynthesis Links C and N Fluxes in C3 Plant Species. PLoS ONE.

[16] Anderegg L.D.L., Loy X., Markham I.P., Elmer C.M., Hovenden M.J., HilleRisLambers J., Mayfield M.M. (2021). Aridity drives coordinated trait shifts but not decreased trait variance across the geographic range of eight Australian trees. New Phytol..

[17] Fajardo A., Piper F. (2011). Intraspecific trait variation and covariation in a widespread tree species (Nothofagus pumilio) in southern Chile. New Phytol..

[18] Zang U., Goisser M., Meyer N., Häberle K.-H., Borken W. (2021). Chemical and morphological response of beech saplings (Fagus sylvatica L.) to an experimental soil drought gradient. For. Ecol. Manag..

[19] Zarzosa P.S., Herraiz A.D., Olmo M., Ruiz-Benito P., Barrón V., Bastias C.C., de la Riva E.G., Villar R. (2021). Linking functional traits with tree growth and forest productivity in Quercus ilex forests along a climatic gradient. Sci. Total. Environ..

[20] Yang Y., Gou R., Li W., Kassout J., Wu J., Wang L., Peng C., Lin G. (2021). Leaf Trait Covariation and Its Controls: A Quantitative Data Analysis Along a Subtropical Elevation Gradient. J. Geophys. Res. Biogeosci..

[21] Gong Y., Ling H., Lv G., Chen Y., Guo Z., Cao J. (2019). Disentangling the influence of aridity and salinity on community functional and phylogenetic diversity in local dryland vegetation. Sci. Total. Environ..

[22] Sun W., Song X., Mu X., Gao P., Wang F., Zhao G. (2015). Spatiotemporal vegetation cover variations associated with climate change and ecological restoration in the Loess Plateau. Agric. For. Meteorol..

[23] Li S., Liang W., Fu B., Lü Y., Fu S., Wang S., Su H. (2016). Vegetation changes in recent large-scale ecological restoration projects and subsequent impact on water resources in China’s Loess Plateau. Sci. Total Environ..

[24] Fu B., Wang S., Liu Y., Liu J., Liang W., Miao C. (2017). Hydrogeomorphic Ecosystem Responses to Natural and Anthropogenic Changes in the Loess Plateau of China. Annu. Rev. Earth Planet. Sci..

[25] Shi H., Shao M. (2000). Soil and water loss from the Loess Plateau in China. J. Arid. Environ..

[26] Cornelissen J.H.C., Lavorel S., Garnier E., Díaz S., Buchmann N., Gurvich D.E., Reich P., ter Steege H., Morgan H.D., Van Der Heijden M.G.A. (2003). A handbook of protocols for standardised and easy measurement of plant functional traits worldwide. Aust. J. Bot..

[27] Wright I.J., Dong N., Maire V., Prentice I.C., Westoby M., Díaz S., Gallagher R.V., Jacobs B.F., Kooyman R., Law E.A. (2017). Global climatic drivers of leaf size. Science.

[28] Mediavilla S., Martínez-Ortega M., Andrés S., Bobo J., Escudero A. (2021). Premature losses of leaf area in response to drought and insect herbivory through a leaf lifespan gradient. J. For. Res..

[29] Reich P., Walters M. (1994). Photosynthesis-nitrogen relations in Amazonian tree species. II. Variation in nitrogen vis-a-vis specific leaf area influences mass-and area-based expressions. Oecologia.

[30] Cui E., Weng E., Yan E., Xia J. (2020). Robust leaf trait relationships across species under global environmental changes. Nat. Commun..

[31] Hu Y., Zuo X., Yue P., Zhao S., Guo X., Li X., Medina-Roldán E. (2020). Increased Precipitation Shapes Relationship between Biochemical and Functional Traits of *Stipa glareosa* in Grass-Dominated Rather than Shrub-Dominated Community in a Desert Steppe. Plants.

[32] De Kauwe M.G., Lin Y.S., Wright I.J., Medlyn B.E., Crous K.Y., Ellsworth D.S., Maire V., Prentice I.C., Atkin O.K., Rogers A. (2016). A test of the ‘one-point method’for estimating maximum carboxylation capacity from field-measured, light-saturated photosynthesis. New Phytol..

[33] Niinemets Ü., Keenan T., Hallik L. (2015). A worldwide analysis of within-canopy variations in leaf structural, chemical and physiological traits across plant functional types. New Phytol..

[34] Taud H., Mas J. (2018). Multilayer perceptron (MLP). Geomatic Approaches for Modeling Land Change Scenarios.

[35] Cornwell W.K., Wright I., Turner J., Maire V., Barbour M.M., Cernusak L., Dawson T., Ellsworth D., Farquhar G.D., Griffiths H. (2018). Climate and soils together regulate photosynthetic carbon isotope discrimination within C3plants worldwide. Glob. Ecol. Biogeogr..

[36] Maire V., Wright I.J., Prentice I.C., Batjes N.H., Bhaskar R., van Bodegom P.M., Cornwell W.K., Ellsworth D., Niinemets U., Ordonez A. (2015). Global effects of soil and climate on leaf photosynthetic traits and rates. Glob. Ecol. Biogeogr..

[37] Yang Y., Wang H., Harrison S.P., Prentice I.C., Wright I.J., Peng C., Lin G. (2019). Quantifying leaf-trait covariation and its controls across climates and biomes. New Phytol..

[38] Oksanen J., Blanchet F., Kindt R., Legendre P., O’hara R., Simpson G., Solymos P., Stevens M., Wagner H. (2020). Multivariate Analysis of Ecological Communities. http://cran.rproject.org/package=vegan.

[39] Legendre P., Legendre L. (2012). Numerical Ecology.

[40] Dong N., Prentice I.C., Wright I.J., Evans B.J., Togashi H.F., Caddy-Retalic S., McInerney F.A., Sparrow B., Leitch E., Lowe A.J. (2020). Components of leaf-trait variation along environmental gradients. New Phytol..

[41] De la Riva E.G., Violle C., Perez-Ramos I.M., Maranon T., Navarro-Fernandez C.M., Olmo M., Villar R. (2018). A Multidimensional Functional Trait Approach Reveals the Imprint of Environmental Stress in Mediterranean Woody Communities. Ecosystems.

[42] Shao J.J., Yuan T.F., Li Z., Li N., Liu H.Y., Bai S.H., Xia J.Y., Lu M., Zhou X.H. (2019). Plant evolutionary history mainly explains the variance in biomass responses to climate warming at a global scale. New Phytol..

[43] Sultan S.E. (2000). Phenotypic plasticity for plant development, function and life history. Trends Plant Sci..

[44] Willmore K.E., Young N.M., Richtsmeier J.T. (2007). Phenotypic Variability: Its Components, Measurement and Underlying Developmental Processes. Evol. Biol..

[45] Karagatzides J.D., Ellison A.M. (2009). Construction Costs, Payback Times, and the Leaf Economics of Carnivorous Plants. Am. J. Bot..

[46] Poorter H., Pepin S., Rijkers T., De Jong Y., Evans J., Körner C. (2005). Construction costs, chemical composition and payback time of high- and low-irradiance leaves. J. Exp. Bot..

[47] Chen J.-L., Reynolds J.F., Harley P.C., Tenhunen J.D. (1993). Coordination theory of leaf nitrogen distribution in a canopy. Oecologia.

[48] Farquhar G., Von Caemmerer S., Berry J.A. (1980). A biochemical model of photosynthetic CO_2_ assimilation in leaves of C3 species. Planta.

[49] Wang H., Prentice I.C., Keenan T.F., Davis T.W., Wright I.J., Cornwell W.K., Evans B.J., Peng C. (2017). Towards a universal model for carbon dioxide uptake by plants. Nat. Plants.

[50] Lambers H., Shane M.W., Cramer M., Pearse S.J., Veneklaas E.J. (2006). Root Structure and Functioning for Efficient Acquisition of Phosphorus: Matching Morphological and Physiological Traits. Ann. Bot..

[51] Fuchs S., Leuschner C., Link R.M., Schuldt B. (2021). Hydraulic variability of three temperate broadleaf tree species along a water availability gradient in central Europe. New Phytol..

[52] Scheiter S., Langan L., Higgins S.I. (2013). Next-generation dynamic global vegetation models: Learning from community ecology. New Phytol..

[53] Harrison S.P., Cramer W., Franklin O., Prentice I.C., Wang H., Brännström Å., de Boer H., Dieckmann U., Joshi J., Keenan T.F. (2021). Eco-evolutionary optimality as a means to improve vegetation and land-surface models. New Phytol..

[54] Sakschewski B., Von Bloh W., Boit A., Poorter L., Peña-Claros M., Heinke J., Thonicke K. (2016). Resilience of Amazon forests emerges from plant trait diversity. Nat. Clim. Chang..

[55] Isbell F., Craven D., Connolly J., Loreau M., Schmid B., Beierkuhnlein C., Bezemer M., Bonin C., Bruelheide H., De Luca E. (2015). Biodiversity increases the resistance of ecosystem productivity to climate extremes. Nature.

